# Experiences of physical activity during pregnancy in Danish nulliparous women with a physically active life before pregnancy. A qualitative study

**DOI:** 10.1186/1471-2393-10-33

**Published:** 2010-06-29

**Authors:** Hanne K Hegaard, Hanne Kjaergaard, Peter P Damm, Kerstin Petersson, Anna-Karin Dykes

**Affiliations:** 1 The Unit of Caring Sciences, Department of Health Sciences, Faculty of Medicine, Lund University, Sweden; 2 JMC Research - Panum, Section 3341, Juliane Marie Centre, Rigshospitalet, Copenhagen University Hospital, Denmark; 3 Department of Obstetrics, Juliane Marie Centre, Rigshospitalet, Copenhagen University Hospital, Denmark; 4 University of Copenhagen, Faculty of Health Sciences, Copenhagen, Denmark; 5 Malmo University, Faculty of Health and Society, Sweden

## Abstract

**Background:**

National guidelines recommend that healthy pregnant women take 30 minutes or more of moderate exercise a day. Most women reduce the level of physical activity during pregnancy but only a few studies of women's experiences of physical activity during pregnancy exist. The aim of the present study was to elucidate experiences and views of leisure time physical activity during pregnancy in nulliparous women who were physically active prior to their pregnancy.

**Methods:**

A qualitative study was conducted by means of personal interviews. Nineteen women, all with a moderate pre-pregnancy level of physical activity but with different levels of physical activity during pregnancy, participated in the study. Content analysis was applied.

**Results:**

In the analyses of experiences and views of physical activities during pregnancy, four categories and nine sub-categories were developed: *Physical activity as a lifestyle *(Habit and Desire to continue), *Body awareness *(Pregnancy-related discomfort, Having a complicated pregnancy and A growing body), *Carefulness *(Feelings of worry and Balancing worry and sense of security) and *Sense of benefit *(Feelings of happiness and Physical well-being).

**Conclusion:**

As other studies have also shown, women find that the discomfort and complications associated with pregnancy, the growing body, and a sense of insecurity with physical activity are barriers to maintaining former levels of physical activity. This study adds a new perspective by describing women's perceptions of these barriers and of overcoming them - thus, when pregnant, the majority of the women do not cease to be physically active but continue to be so. Barriers are overcome by applying one's own experience, looking to role models, mirroring the activities of other pregnant women and following the advice of experts (midwives/physiotherapists). Women then continue to be physically active during pregnancy, most often to a lesser extent or in alternative activities, and derive considerable enjoyment and physical well-being from this.

## Background

Today several national guidelines recommend that pregnant women take 30 minutes or more of moderate exercise on most, if not all, days of the week in the absence of either medical or obstetric complications [1,2]. Moderate exercise is described as brisk walking [3]. Many studies have shown however, that there seems to be a discrepancy between these recommendations and what women actually do [4,5].

In a cross-sectional study less than a fifth followed the recommendations of moderate exercise during pregnancy [5] and follow-up studies found that women engaged in more leisure time physical activities prior to than during pregnancy [4,6]. When comparing exercise before and during pregnancy with regard to frequency, duration and intensity, a reduction in all variables throughout pregnancy is shown [6-8]. The main reasons for not exercising were tiredness, feeling unwell, being too busy and, in late pregnancy, exercise being uncomfortable [9]. The concern that certain sports might be hazardous to the pregnancy may also affect exercise levels [9]. Lack of time, lack of energy, concern for the baby and discomfort caused by activity have also been mentioned as barriers to exercise [10]. One study reported that women reduced their exercise level due to physical limitations, less motivation or a perceived risk, depending on the activity [11]. Another study found that the primary reasons for exercising during pregnancy were for fitness and enjoyment.

Researchers have studied enablers/barriers to continue exercise during pregnancy [10] and women's beliefs about exercising during their pregnancy in an attempt to increase women's exercise behaviour during their childbearing years [12]. The latter is relevant as epidemiological studies have shown an association between maintenance of pre-pregnancy level of physical activity during pregnancy and reduced risk of gestational diabetes and pre-eclampsia [13,14]. Factors and experiences, which may increase exercise during pregnancy, could be examined in studies with qualitative designs, however few such studies exist. The topic of nulliparas' experiences and views of leisure time physical activity during pregnancy is described only scantly in other studies.

The aim of this study was to elucidate experiences and views of leisure time physical activity during pregnancy in nulliparous women who were physically active before pregnancy.

## Methods

Women were selected for invitation to the study among participants in the Danish Dystocia Study, a multi centre cohort study comprising 4800 nulliparous women in 2004-2005 [15]. Participation in the Danish Dystocia Study involved completing a self-administered questionnaire at 37 weeks of gestation on maternal and socio-demographic characteristics, lifestyle factors and fertility history. In the questionnaire, the women were asked: "When you look back on the year before you became pregnant, which would you say is the most appropriate description of your activities?:

1. reading, watching television, or pursuing some other sedentary occupation;

2. walking, cycling, or other light exercise at least four hours a week (including Sunday walks, light gardening, and cycling or walking to work);

3. sports or heavy gardening at least four hours a week;

4. hard training and competing in sports regularly and several times a week.

Leisure time activity was thus categorized as sedentary, light, moderate-to-heavy, or competitive sports. The items of leisure time activities under these four categories were originally developed and validated by Saltin [16,17]. Participants in the present qualitative study were sampled among those subjects who were classified as being physically active in the "moderate-to-heavy" category. The questionnaire also comprised information on the level of physical activity during first, second and third trimester. In order to have a wide sample of information we included women who continued to have a moderate-to-heavy exercise level during first and second trimester but also some women who lowered this level to the category "light or sedentary" during first and second trimester. We furthermore chose women with and without chronic diseases, women <25 years, 25-35 years and >35 years, non-smokers and smokers, women with BMI <18.5 kg/m^2^, 18.5 kg/m^2 - ^25 kg/m^2 ^and >25 kg/m^2^, and women who had completed lower, intermediate and higher education (Table 1). Potential participants were contacted by letter and provided with written information about the study, including its aim. Subsequently, they were contacted by telephone and asked to participate in the present interview study on views and experiences regarding physical activity prior to and during pregnancy with their fist child. A convenience sample of 20 women was chosen [18]. In total, 20 women were contacted by telephone, all of whom wished to participate. One person subsequently withdrew due to illness. The interval between the birth of the first child and the interview varied from three to four years. All interviews took place from February to July 2008.

**Table 1:** Participants in relation to maternal, socio-demographic characteristics and lifestyle factors gathered by self-administered questionnaires during pregnancy (n = 19)

Socio-demographic and life factors	N
Leisure time physical activity during first and second trimester*	
Sedentary	6
Light	7
Moderate-to-heavy	6
Competitive	0

**Age (years)**	
<25	1
25-29	8
30-34	6
35+	4

**Pre-pregnancy BMI (kg/m^2^)**	
<18.5	1
18.5-25	15
25+	3

**Fertility treatment**	
Yes	2
No	17

**Chronic diseases or conditions**	
Yes	2
No	17

**Smoking habits before pregnancy**	
Non-smoker	13
Smoker	6

**Years of education**	
10-11	4
12	14
Other	1

*Sedentary: reading, watching television, or pursuing other sedentary occupations;

**Light**: walking, cycling, or other light exercise at least four hours a week (including Sunday walks, light gardening, and cycling or walking to work);

**Moderate-to-heavy**: sports or heavy gardening at least four hours a week;

**Competitive**: heavy training and competitive sports regularly and several times a week (16).

The study protocol was presented to the Research Ethics Committee of Copenhagen, which had no objections to its implementation ((KF) 07-00-010/04). Informed consent was procured from all participants, and it was stressed that they were free to withdraw from the study at any time. The Helsinki Declaration was followed during both the data collection and data analysis phases [19].

## Data collection

This study aimed to collect knowledge about women's own experiences and perceptions of their physical activity during pregnancy. Such information can be gathered through interviews [20]. A thematic interview guide with open-ended questions was developed. The interview guide comprised the following themes: pre-pregnancy physical activity, motives for pre-pregnancy physical activity, what happened upon becoming pregnant, motives for being physically active during pregnancy and the importance of physical activity during pregnancy. The themes were introduced if the informants did not spontaneously address them. All the informants were asked by way of introduction to describe their physical activity from childhood until their pregnancy, and were then asked: "What happened when you became pregnant?" Where relevant they were encouraged to elaborate on their experiences and views. Data on physical activity prior to pregnancy will be presented in a second article. It was emphasized that the interview dealt only with the period before and during pregnancy with their first child. Depending on her preference, the interview took place in the woman's home, at her place of work or in the research section of Rigshospitalet. The interviews were conducted according to Kvale [18] and were all undertaken by the first author (HKH). The interviews were audio-taped and transcribed verbatim including non-verbal expressions such as laughter and silence. In total, 19 women were interviewed, 14 in their homes, two at their places of work, and three in the research section of Rigshospitalet. The duration of the interviews varied from 15 to 50 minutes.

### Data analysis

Few studies of women's experiences of physical activity during pregnancy exist. As no predetermined theoretical framework structure existed on this topic, an inductive analysis approach was relevant [20,21].

Content analysis has been applied in previous studies on pregnancy and sports [22,23], and Burnard's 14 steps content analysis was used [24], with the exception of step 11 which concerns informant checking. Immediately after each interview notes about the topics were taken. Transcripts were read through repeatedly in order to become fully familiar with the individual informant's situation and to get a feeling for the whole. Subsequently the transcripts were read through line by line and all meaning units were given a code, a phase called "open coding". Four authors (HKH, HK, KP and AKD) then read and coded two interviews independently and the four sets of codes were compared and discussed. As the codes were almost identical consensus was easily reached. Following this, a further seven interviews were coded separately by two authors (HKH and either HK, AKD or KP). The codes were compared and found to be almost identical. The open coding of all the interviews resulted in 675 codes. For each of the 19 interviews, the first author then clustered similar codes in sub-categories and categories. After carefully having gone through all the interviews, the list of codes for each interview was re-worked and nine sub-categories and four categories were generated. The transcripts were then re-read to ensure that the categories of each interview covered all aspects of the interview, and each interview was put into a coding scheme with defined text units, codes, sub-categories and categories. The next step was to aggregate and print each of the four categories with the accompanying sub-category, codes and copies of the defined text units (Table 2). A control was undertaken by the other authors to ensure conformity between the defined text unit and the categories. The section "Results" was written on the basis of the content of the categories and reference was made to the original text with the supporting quote. Throughout this process we reverted to and re-read the transcribed material in any case of ambiguity. The final step was the discussion.

**Table 2:** Overview of analyses, examples

Quotes	Codes	Sub-categories	Categories
I:19: because I went there, I think I went there a whole year before I got pregnant HH: yes I:19: and so it was natural for me to continue, but I combined it with jogging HH: yes I:19: and then I stopped jogging and continued down there at a lower pace	Natural to continue Stopped jogging Continued at a lower pace	Habit Desire to continue	**Physical activity as a lifestyle**

I:2: I mean it's limited what you can do with such a big stomach towards the end, right? HH: yes I:2: and then it is really nice just to bicycle for about 20 minutes or so	Limited what you can do with such a stomach, so it is nice to bicycle	A growing body	**Body awareness**

I:14: well I was a little bit careful, um, I wasn't really totally comfortable with it, so I didn't exert myself as much as, um, I otherwise would have, or did before HH: yes I:14: I didn't go to it quite as much and I wasn't as out of breath when I was done	Was more careful as I didn't feel secure I didn't go to it quite as much	Balancing worry and sense of security	**Carefulness**

I:5: yes, and I could feel that I could do it, so, well, I was happy and full of energy and I had no pain anywhere, didn't have any problems,	Could feel that I could do it, happy, full of energy and had no pain anywhere (I:5)	Feelings of happiness	**Sense of benefit**

In qualitative research it is particularly important to address the authors' potential pre-understanding of the topic [20], in order not to compromise the research. Before initiating this study, pre-understanding was described and discussed among the authors, whose pre-understanding was based on knowledge gained from research into pregnancy and physical activity as well as on experience in clinical practice (midwife, obstetrician and paediatric nurse).

### Rigour

Trustworthiness is an important concept in qualitative studies, comprising elements such as credibility, dependability and transferability [25]. Credibility (the true value) implies inter alia that the selection of participants with diverse experiences affords a greater opportunity to elucidate the research topic from various aspects. We have attempted this in our study by selecting women having the same level of physical activity prior to pregnancy but varying levels of physical activity during pregnancy. As no new sub-categories appeared in the latest analyses, this would seem to indicate that the number of informants was sufficient. A discussion within the research group of the coding, sub-categories and main categories was also a means of enhancing credibility. Dependability concerns the extent to which data change over time, and whether the authors' decisions change over time. We have tried to take this into account by requesting responses from all participants to the same introductory question and the same themes in the interview guide and, through discussion, reaching agreement among the authors as to coding, sub-categories and categories.

## Results

In total 19 women were interviewed. The four categories generated were: *Physical activity as a lifestyle, Body awareness, Carefulness*, and *Sense of benefit*.

### Physical activity as a lifestyle

The sub-categories in this category were: *Habit *and *Desire to continue*. The majority of the women felt that their physically active lifestyle prior to pregnancy influenced their physical activity during pregnancy. Training at fitness centers, jogging, and cycling to and from work, as most did prior to pregnancy, are described by the majority of the women as activities, which were maintained as a matter of course during pregnancy. The retention of a physically active life in pregnancy was perceived by the women not as a deliberate but rather as an unconscious continuation of their habits and daily life. Most of the women found that their physically active lives continued at a pace lower than the pre-pregnancy level, although some exercised more.

"I felt it was just part of my daily routine and part of my life, so I simply went on with it (I:4)

Some of the women deliberately chose to carry on with their physical activity despite their pregnancy, and were of the opinion that physical activity should not be discontinued simply because they were pregnant. Pregnancy was not considered a disease by some of the women, who continued to be physically active. A number of the interviewed women had observed that other expecting mothers could exercise during their pregnancy and were themselves thus also motivated to continue exercising during their own pregnancies. The women related that they wished to continue to exercise in order not to give up the sense of well being that this afforded them - likewise, they wished to keep in shape during pregnancy. One expressed the view that it would be depressing to have to start from scratch upon resuming training after pregnancy. Several were aware that physical activity for a half hour each day was recommended, and that physical fitness contributed to an easier pregnancy and labour and helped avoid excessive weight gain during pregnancy.

"I was quite determined that my pregnancy should not stop me from being physically active... because I have always enjoyed exercising and I was sure that everything would go much better if I were physically active (I:5)

Several women felt that they could maintain their exercise patterns during pregnancy, while others found they were able to exercise less than expected or had to switch to new activities. The latter were described as yoga, prenatal gymnastics or swimming, all of which were seen to be activities that could be undertaken during pregnancy. The majority of the women described their observation that their routine of bicycling was something they could continue throughout their pregnancy.

"I bicycled as much as I could because it didn't bother me to cycle, I always could, meaning that I could right up to the very end (I:14)

The women who suffered psychologically challenging events such as the death of a parent or estrangement from the child's father perceived that they did not have the energy or desire for more physically intensive activities. They gave them up for a while or for the remainder of the pregnancy.

"and if I let myself become psychologically upset, then I lose the motivation to exercise, and that started with my daughter's father (I:16)

### Body awareness

The issue of body awareness included the sub-categories: *Pregnancy-related discomfort, Having a complicated pregnancy *and *A growing body*. Bodily changes could affect physical activity both early and late in the pregnancy and are experienced as barriers to physical activity. Early pregnancy discomfort such as nausea or fatigue was seen by some women as grounds to give up the long bicycle rides to and from work and workouts in the fitness center. Nausea is seen as causing an aversion to being with other perspiring people at fitness training. Cycling or walks in the open air are, on the other hand, perceived to diminish nausea. One individual felt so poorly when training at a fitness center that she was forced to refrain from training even before the pregnancy was ascertained. She found this discouraging.

"just before I discovered that I was pregnant I was at a spinning class with my husband where at the end of the class I felt so awful, worse than I have ever experienced"... I simply couldn't go on" (I:1)

Physiological reactions such as pressure on the bladder while jogging or the frequent need to urinate during aerobics were perceived as unpleasant and reasons to switch to other forms of exercise. One woman's appetite when exercising was so great that she became unwell. She found however, that this could be avoided if she ate while doing prenatal gymnastics.

Pregnancy complications such as bleeding, emesis, after effects of fertility treatment or large uterine fibroids made some women to feel unable to be physically active during part of the pregnancy and one woman during the entire pregnancy. Several later resumed physical activity, taking up more gentle forms of exercise such as swimming, prenatal gymnastics, cycling or slow walks.

"Even though I was physically active before I became pregnant, and worked out, I would guess, 3-4 times a week... I hardly trained at all... I didn't have the energy or strength for it at all (I:1)

The fact that the body grows during pregnancy is perceived to affect both the intensity and preference of physical activity. Pregnant women who jogged felt that this became both heavier and harder even before their pregnancy became visible, and they had to reduce both pace and distance. Several could continue on stationary bicycles but not on spinning bicycles, as there was no space for the abdomen. These women also found that they could replace the spinning bicycle with the cross-trainer. The cross-trainer functioned well as there was space for the pregnant abdomen and it did not cause abrupt jolts of the body as did the treadmill or jogging in open terrain. Training in a fitness center was perceived as beneficial as the women could make individual modifications in their training program to adapt it to their changing bodies. Strength training became difficult however, towards the end of the pregnancy, as there was no space in the machines for the enlarging abdomen, but the women could then take up other activities.

"used the cross-trainer a lot, where you stand with your arms and legs, I mean, I think it's brilliant, because there is room for your stomach" (I:8)

"Well, I think two weeks before I gave birth I was of course working out a bit less, towards the end you can't sit at the machines with such a big stomach, but you can still do the Power Walker or stand on a stepper for 10 minutes or something, right?" (I:2)

Their large, fecund, growing bodies could psychologically discourage the pregnant women from training, as they felt fat and exposed in a fitness class with non-pregnant women. The growing body was perceived as demotivating, as exercise is normally taken to get fit and into better shape, while during pregnancy the opposite occurs, even with exercise.

"because you just get bigger even though you work out, so I think that can be a real demotivator or whatever you call it (I:19)

The growing stomach was perceived as problematic towards the end of the pregnancy if the women cycled on a racing or mountain bike, as their knees knocked against their abdomen. Pregnant women having bicycles with upright handlebars did not encounter these problems. Women did find, however, that they could continue to bicycle if they purchased or borrowed a bicycle with upright handlebars where there was room for their stomach. Some did not discover this and stopped bicycling. The pregnant women who continued to bicycle found cycling preferable to walking or transportation by bus.

"so I had problems because I had to lean forward so much on my bike, so at the end when I got other handlebars put on and later exchanged bikes with a friend who had one of these old-fashioned bikes, then I could sit upright so I could bike" (I:11)

### Carefulness

Sub-categories were: *Feelings of worry *and *Balancing worry and sense of security*, the latter with the sub sub-categories *Security from within *and *Security from without*. All the pregnant women experienced a certain worry about being physically active during pregnancy; this influenced the level of activity to varying degrees and could be experienced as a barrier to continuing physical activity. Most concerned were those pregnant women who had received fertility treatment or had themselves previously miscarried or knew women who had miscarried. Women were afraid that physical activity might lead to miscarriage. For this reason they were cautious, one woman remaining inactive throughout her entire pregnancy.

"but before I had my son I had a miscarriage, so I had sort of a period where I didn't dare dance as wildly again while I was expecting my son, so I was especially careful (I:13)

"so it was that, really, now I was pregnant, like we were under treatment to get pregnant with IUI, so maybe it was sort of like, now finally we were successful so you didn't want to do too much, in case something went wrong, you know (I:17)

Anxiety and concern were also felt in relation to specific kinds of exercise. Several women refrained from strength training and jogging, considering these kinds of exercise too strenuous during pregnancy. Some women felt an unpleasant heaviness when jogging, a sensation that lead them to fear a miscarriage.

"I had been jogging and the next time I was supposed to go jogging, suddenly I had such an unpleasant heavy feeling, there was this feeling of pressure, and where I thought oh no, I wonder if I am having a miscarriage... but I just felt that heaviness every time I hit the ground with my legs, so I quit jogging" (I:15)

Although all women experienced worry about physical activity during pregnancy, most chose to continue to be active. The women opted to modify their physical activity or switch to a new form of exercise. They subsequently felt reassured with their choice, a reassurance that may be described as coming from within or as coming from without. The few pregnant women who maintained physical activity at a high level related that they believed they could sense what was right and not right for their bodies and thus felt a reassurance that came from within.

"no, well I did sort of feel that I would be able to sense from my body if something was wrong" (I:6)

The two pregnant women who had miscarried reduced their activity or switched to less intensive activities such as swimming, walking or yoga, and felt secure in their decision. One relates how her own inner sense of security was more important than what others told her.

"but I stayed away from jogging, and I stayed away from, I mean I did, I think I did some good things which I had good experiences with, I mean I went to pregnancy yoga, I swam and I cycled and I did some dance training" (I:10)

Expecting mothers also found that a sense of security in being physically active could come from without through professionals and other pregnant women. Those who continued to lift weights were all given special guidance. It is regarded as reassuring to be able to consult professionals and follow exercise programs where consideration is taken of the pregnancy. Some found that the trainer, due to the risk of miscarriage, wanted to postpone resumption of training until after week 12.

"I think we started by waiting the 12 weeks, and then I got a program that worked for me... not too strenuous" (I:2)

Others sensed insecurity in fitness, strength training or jogging and these women chose instead to join specific prenatal exercise classes. This was perceived as generating a sense of security, as the classes were led by professionals whom they considered capable of determining what a pregnant woman may do, and because other pregnant women performed the same physical activity.

"in the training center that had exercise sessions for pregnant women, it was as if it was within the pregnancy limits, there they knew how much you could take...I mean they were aware that they were dealing with pregnant women" (I:3)

One individual had received conflicting information from midwives about physical activity, one of whom expressed the opinion that it was hazardous to the child if the woman's heart rate became too high, while others claimed that this was incorrect. In other cases the pregnant woman found that the midwife, during their conversations, reassured her that it was in order to continue aerobics or begin swimming.

"but then I preferred to take it a bit easier and such, but then my midwife said that it was okay to swim, that it was the best thing to do" (I:12)

### Sense of benefit

This category contains two sub-categories: *Feelings of happiness *and *Physical well-being*. Women who continued to exercise during pregnancy experienced that physical activity had contributed to a wonderful pregnancy both physically and mentally.

Superlatives are prevalent as women relate how they experienced their physical activities during pregnancy. They found it gave more pleasure, well-being, energy and lightheartedness to be physically active. It was described as enjoyable to use one's body, big though it was. It was regarded as a psychological boost to be able to be more active than one anticipated.

"just being active, it gave me energy and made me happy, it made me wildly happy" (I:4)

Yoga as an activity is perceived as pleasurable, relaxing and meditative for the women who tried it, and is described as a good way to cope with the distended pregnant body. Jogging is described by some as pleasant both early in the pregnancy when the breasts were sore but also later in the pregnancy when the stomach was heavy - others did not share this experience. Strength training is described as wonderful. Swimming was found to be the most satisfying form of exercise and was described as pleasant, fantastic and great during pregnancy, even though many of the women did not actually care for swimming prior to their pregnancy.

"but it has been fantastic, and I'm not otherwise much for swimming, but um, it got me into the water anyway" (I:5)

The pregnant women were happy to be active during pregnancy. Several pushed themselves to increase their heart rate, which was experienced as feeling good. Physical activity was perceived to reduce weight gain and have a positive impact on aches in the back and lumbar region. The perception was that jogging could mitigate the sensation that "one's body was a big lump". While swimming, the woman felt that she could use her body as she did before her pregnancy - as almost the only form of exercise to do so. Swimming could, for a while, allow the woman feel a lightness and weightlessness and unburdened by her heavy body. It was pleasant to lie on one's stomach in the water, which wasn't possible on land. It was good to feel the tiredness following a swim. Swimming was perceived to improve blood circulation in the body and partly alleviate edema in the legs. Feelings of discomfort when swimming were also expressed. One woman described that she did not feel it was pleasant to swim in the last part of her pregnancy. She felt she was drowning and was afraid of drowning.

"it was pleasant to for example lie face down when doing the breast stroke, because for a long time it wasn't possible to lie on your stomach" (I:10)

"being out of the water felt heavy and cumbersome towards the end and in the water it just felt like you weighed 50 kilos again" (I:12)

## Discussion

### Modifying physical activity during pregnancy

In our study nearly all women altered their pattern of physical activity during pregnancy and that this is perceived as undertaken in order to adapt to both psychological and physiological factors during pregnancy. This modification is for the majority of the women made by replacing strenuous activities such as jogging and strength training with moderate activities such as swimming and gymnastics. Substituting strenuous activities with more moderate activities during pregnancy is also mentioned in other studies [7,8,26]. The majority of the women relate that they can continue to bicycle if they have upright handlebars. Other studies describe the proportion of pregnant women who bicycle as declining considerably during pregnancy [7,8]. In our study we illustrate that few participants increased the degree of physical activity from a pre-existing high level. This is also shown in earlier studies [27,28].

### Stimulation of physical activity during pregnancy

Having a physically active lifestyle prior to pregnancy is perceived by the majority in this study to promote a woman's physical activity during pregnancy, which is in line with other studies [27-29]. The vast majority of the women in our study had a desire to maintain this activity during pregnancy, believed they could do so, and did so, if possible from a health perspective. This corresponds to results from Hinton's study [27] showing that exercise-self-efficacy, "the belief in one's ability to exercise", is an important determinant for physical activity during pregnancy. The participants relate that the motivation for continuing training was: to maintain fitness, a sense of well-being, and enjoyment of training, reasons which are also found in other studies [9,12]. Such expectations were met, as our active pregnant women found great pleasure and physical well-being in being physically active. Furthermore, the majority of the pregnant women described, that a reason to continue physical active during pregnancy was that they desired to avoid excessive weight gain. Many of the physically active women experienced an acceptable weight gain. A higher maternal weight gain among inactive as compared to active pregnant women was also seen in an epidemiologic study [7]. The pregnant women generally found physical activity to be very beneficial to the mind and the body, alleviating aches in the back and lumbar region as well as edemas, as has been described earlier [30,31].

### Barriers to leisure time physical activity during pregnancy

In accordant with a previous study [10] the present study describes how the complications of pregnancy can prohibit physical activity, and elucidates why not all expecting mothers are able to be physically active. As in other studies [9,10] the discomforts of pregnancy such as fatigue and nausea, and the growing body and thus the cumbersomeness of training, are seen as reasons to stop or cut back training. In our study we find that all of the pregnant women feel a certain degree of worry that participation in physical activity, particularly strenuous activity, may cause miscarriage or in other ways be harmful. Especially women who have miscarried previous pregnancies, have had artificial insemination or know friends who have miscarried experience this apprehension. Concern for the health of the child or a previous miscarriage is described in this study and in others [11,28] as a reason not to be physically active. When jogging, women experience a disagreeable feeling of pressure, and some are afraid that this may lead to miscarriage - leading the majority of pregnant women to discontinue jogging during pregnancy. It is vigorous activities such as jogging that are perceived by other pregnant women as unsafe during pregnancy [9]. In an epidemiological study, women who were physically active in the sense of jogging etc., had a higher risk of aborting than did women who were not physically active [32].

### Overcoming barriers to physical activity

Several studies describe the barriers faced by pregnant women to being physically active during pregnancy [9-11,28]. In our study an entirely new dimension arises in that women relate their experiences in overcoming these barriers in order to continue to be physically active during their pregnancy. Women encounter barriers such as nausea, confinement to bed with hyperemesis, forward-leaning bicycles, discomfort in jogging and strength training and yet they are capable of staying physically active because - unless they have pregnancy-related complications - they are able to switch to another activity. They do so on the basis of their own experience, but also following the example of others, the impetus seeming to be the desire to continue to be active - i.e. as a matter of habit or lifestyle. The intention to continue to be physically active during pregnancy is associated with physical activity during pregnancy [12]. Although physical activity during pregnancy is associated with worry and concern, most pregnant women nevertheless choose to be physically active. Some relate that they have an inner sense of security, which allows them to judge whether it is safe to continue the same activity or whether it is better to replace intensive activity with more moderate activities. Other pregnant women likewise continue their physical activities out of a sense of security, which seems rather to have external origins. Security from without may be described as the security they feel in receiving professional instruction at training centers, attending prenatal exercise classes or being counseled by midwives who are instructors or advise them during consultation. It seems that some pregnant women need help in finding a sense of security in being physically active during pregnancy. As far as is known, this constitutes new knowledge as to how pregnant women manage their insecurity/security so they may continue to be active.

### Clinical implications: The role of midwives and physicians in prenatal care

Because uneasiness with physical activity is a key subject for pregnant women, midwives and physicians must address this in a way that allows the woman to determine how she can feel safe in being physically active. Midwives must ensure that pregnant women are counseled in modifying their activities, including such practical aspects as bicycle handlebars. As prenatal exercise classes are seen as a good type of activity during pregnancy these should be offered to all. Finally, midwives should be aware that some may find it disappointing to be unable to be physically active during their pregnancy.

### Limitations

A limitation in this study is that women were interviewed 3-4 years after the birth of their first child, which could introduce risk of recall bias. Nevertheless, studies have shown that women's memories of experiences with their first birth are very accurate after more than 20 years [33] and that accurate perinatal information can generally be obtained with a recall period from four to six years [34]. The level of detail in the women's descriptions of their experiences in this study likewise indicates that women clearly recall their level of exercise during their first pregnancy.

Transferability refers applicability of findings to other contexts. The experiences of leisure time physical activity appear to be experiences that women in other western societies can share. There is no reason to believe that the same findings would not be ascertained in a group of women having the same characteristics [35].

Finally, the assessment of physical activity relied on self-reporting and the questions concerning physical activity has not been validated in pregnant women [16,17].

## Conclusions

The majority of the women with a physically active lifestyle prior to pregnancy continue this lifestyle into pregnancy, as it is a habit and because of a great desire to maintain it. The challenge facing the woman is that the mind and the body react differently than before pregnancy. Pregnancy is a new state in which discomfort, complications, the growing body or uneasiness with physical activity are perceived by the majority of the women as barriers to physical activity. The majority of women overcome these barriers through their own experience, role models, mirroring other pregnant women, and the counsel of experts (midwives/physiotherapists) and continue their physical activity, simply at a lower level, through other activities or with other equipment, and derive much pleasure and physical well-being from doing so. Pathological conditions or excessive worry about physical activity can lead to inactivity. To our knowledge these findings as to how some women overcome these barriers to physical activity during pregnancy have not been previously described. Therefore, further research is needed to supplement and validate our findings.

## Competing interests

The authors declare that they have no competing interests.

## Authors' contributions

HKH, AKD, HK and KP planned this qualitative study. HKH carried out data collection. All five authors participated in the analyses. HKH wrote the drafts of this manuscript, which HK, PD, KP and AKD commented on. All five authors read and approved the final manuscript.

## Pre-publication history

The pre-publication history for this paper can be accessed here:

http://www.biomedcentral.com/1471-2393/10/33/prepub
